# Relationship between Serum Vitamin D and Leg Strength in Older Adults with Pre-Dialysis Chronic Kidney Disease: A Preliminary Study

**DOI:** 10.3390/ijerph17041433

**Published:** 2020-02-23

**Authors:** Akira Saito, Koji Hiraki, Yuhei Otobe, Kazuhiro P. Izawa, Tsutomu Sakurada, Yugo Shibagaki

**Affiliations:** 1 Department of Rehabilitation Center, St. Marianna University School of Medicine Hospital, 2-16-1 Sugao, Miyamae-ku, Kawasaki-city, Kanagawa 216-8511, Japan; akira.saito@marianna-u.ac.jp; 2 Department of Rehabilitation Medicine, Kawasaki Municipal Tama Hospital,1-30-37 Syukugawara Tama-ku Kawasaki-city, Kanagawa 214-8525, Japan; y-oto@marianna-u.ac.jp; 3 Department of Public Health, Graduate School of Health Sciences, Kobe University, Kobe-city, Hyogo 654-0142, Japan; izawapk@harbor.kobe-u.ac.jp; 4 Division of Nephrology and Hypertension, Department of Internal Medicine, St. Marianna University School of Medicine Hospital, 2-16-1 Sugao, Miyamae-ku, Kawasaki-city, Kanagawa 216-8511, Japan; sakurada@marianna-u.ac.jp (T.S.); shibagaki@marianna-u.ac.jp (Y.S.)

**Keywords:** serum vitamin D, leg strength, older adults, pre-dialysis chronic kidney disease

## Abstract

Active vitamin D (calcitriol, or 1.25 (OH) 2 D) is associated with muscle weakness, falls, and fracture in community-dwelling older people. This study aimed to investigate the relationship between a serum active vitamin D level and lower extremity muscle strength in elderly patients with pre-dialysis chronic kidney disease (CKD). This cross-sectional study included 231 patients with CKD treated conservatively as outpatients. We analyzed patient background factors, including age, sex, body mass index (BMI), intact parathyroid hormone (PTH), phosphorus, calcium, albumin, serum calcitriol level as an indicator of active vitamin D, and estimated glomerular filtration rate (eGFR) collected from medical records. As an index of lower extremity muscle strength, the isometric knee extension muscle strength-to-weight ratio (kgf/kg) was calculated. The mean patient age was 75.9 ± 6.1 years (68.8% male), and the BMI was 24.1 ± 3.8 kg/m^2^. A significant correlation was observed between knee extensor muscle strength and serum calcitriol level (*r* = 0.32, *p* < 0.01), age (*r* = –0.30, *p* < 0.01), BMI (r = −0.31, *p* < 0.01), intact PTH (*r* = −0.22, *p* < 0.01), phosphorus (*r* = −0.29, *p* < 0.01), albumin (*r* = −0.28, *p* < 0.01), and eGFR (*r* = 0.25, *p* < 0.01). Multiple regression analysis showed calcitriol to be significantly associated with knee extensor muscle strength (β: 0.14, 95% confidence interval: 0–0.002, *p* = 0.04) after adjustment for covariates. These results suggest that the serum active vitamin D level is associated with lower extremity muscle strength in older adults with pre-dialysis CKD. It is necessary to verify whether vitamin D supplementation increases lower extremity muscle strength in pre-dialysis CKD patients.

## 1. Introduction

Chronic kidney disease (CKD) is a risk factor for cerebral and cardiovascular disease. In Japan, 13.3 million are estimated to suffer from CKD [1], and among end-stage CKD patients, many progress to dialysis [2].

As the CKD progresses, various types of CKD–Mineral and Bone Disorder (CKD–MBD) are caused by a decrease in active vitamin D (calcitriol, or 1.25-dihydroxy-vitamin D (1.25 (OH) 2 D)) and the accumulation of phosphorus. CKD–MBD is a concept originally proposed to occur in the dialysis phase, but its pathology has already been known to occur from the pre-dialysis phase [3]. Bone fracture as well as vascular calcification are the main clinical problems of CKD–MBD. Since bone fracture typically occurs from falls, and patients with CKD were at a high risk of falls due to compromised physical function and sarcopenia, it is important to maintain physical function in order to prevent bone fractures.

Vitamin D is also associated with muscle weakness, falls, and fracture in community-dwelling older people [4,5]. Romy et al. report that vitamin D deficiency results in reduced muscle mass and reduced physical performance [6]. The mechanism by which the vitamin D status affects muscle metabolism and muscle function has not been fully elucidated [7]. However, in patients with pre-dialysis CKD, the relationship between active vitamin D and lower extremity muscle strength is unknown.

The purpose of this study was to investigate the relationship between the serum calcitriol level and lower extremity muscle strength in elderly patients with pre-dialysis CKD.

## 2. Methods

### 2.1. Patients

A total of 231 study participants were patients with CKD treated conservatively in the outpatient clinics of the Division of Nephrology and Hypertension, St. Marianna University Hospital, from January 2011 to April 2019. Exclusion criteria were patients under 65 years of age, those with missing values in the survey items, those with motor paralysis and severe bone and joint disease, and those with dementia who had difficulty understanding instructions.

### 2.2. Measurements

This was a cross-sectional study in which patient background factors, including age, sex, body mass index (BMI), intact parathyroid hormone (PTH), phosphorus, calcium, albumin, serum calcitriol level as an indicator of active vitamin D, and estimated glomerular filtration rate (eGFR) were collected from medical records and analyzed.

Moreover, as an index of lower extremity muscle strength, the isometric knee extension muscle strength-to-weight ratio (kgf/kg) of the lower leg as measured with the patient sitting in a chair was calculated. Strength measurements were performed twice on the left and right sides [8] using a hand-held μ-TAS MT-1 dynamometer (Anima Co. Ltd., Tokyo, Japan).

The maximum value measured was used as the indicator of muscle strength. We calculated the muscle strength-to-weight ratio value by dividing the maximum muscle strength value (kgf) by the body weight (kg) as described in a previous study [8].

### 2.3. Statistical Analysis

The results are expressed as the mean ± standard deviation. We used the Spearman rank correlation coefficient to determine the correlation between the strength-to-weight ratio and each survey item. Subsequently, in clarifying the relationship between the lower extremity muscle strength and the calcitriol level, multiple regression analysis (forced input method) was performed using the adjusted factors as the adjustment variables.

The overall level of statistical significance was set at 0.05. All statistical analyses were performed using the SPSS version 21 J software program (IBM, SPSS, Tokyo, Japan).

The present study was approved by the St. Marianna University Institutional Committee on Human Research. Written informed consent was obtained from each patient.

## 3. Results

Of the 231 patients, 159 (68.8%) patients were male. The mean patient age was 75.9 ± 6.1 years, and the BMI was 24.1 ± 3.8 kg/m^2^. Serum calcitriol, eGFR, intact PTH, phosphorus, calcium, albumin, and the index of knee extension muscle strength are shown in Table 1.

**Figure 1:**
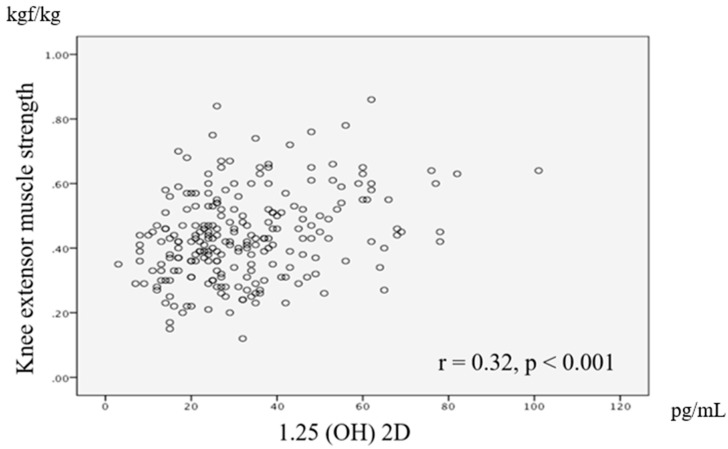
The relationship between knee extensor muscle strength and active vitamin D (calcitriol, or 1.25 (OH) 2 D) using the Spearman rank correlation coefficient. There was a significant positive correlation between leg strength and 1.25 (OH) 2 D (*r* = 0.32, *p* < 0.01).

A significant correlation was observed between knee extensor muscle strength and serum calcitriol level (*r* = 0.32, *p* < 0.01), age (*r* = −0.30, *p* < 0.01), BMI (*r* = −0.31, *p* < 0.01), intact PTH (*r* = −0.22, *p* < 0.01), phosphorus (*r* = −0.29, *p* < 0.01), albumin (*r* = −0.28, *p* < 0.01), and eGFR (*r* = 0.25, *p* < 0.01). We also found a significant correlation between the knee extension muscle strength-to-weight ratio as a dependent variable and the calcitriol level as an independent variable. A scatter chart of the relationship between knee extension muscle strength-to-weight ratio and the calcitriol level is shown in Figure 1.

The results of multiple regression analysis showed the serum calcitriol level to be significantly associated with knee extensor muscle strength (β: 0.14, 95% confidence interval: 0–0.002, *p* = 0.04) after adjustment for covariates.

## 4. Discussion

To our knowledge, this is the first report to identify a relationship between active vitamin D measured by serum calcitriol and knee extensor muscle strength in elderly patients with pre-dialysis CKD. These results indicate that calcitriol may be one of the factors related to knee extensor muscle strength in these patients. After adjustment for patients’ characteristics, including age, sex, BMI, Intact PTH, Phosphorus, Albumin, eGFR, and calcitriol level was still associated with knee extensor muscle strength.

### 4.1. The Relationship between Knee Extensor Muscle Strength and Active Vitamin D

There is a vitamin D receptor (VDR) in the muscle. Abnormal muscle differentiation is thought to be antagonized through the VDR [9,10]. In addition, the VDR contributes to the normalization of calcium metabolism related to muscle tone and muscle contraction [9].

An anti-aging hormone called klotho was recently reported [11] that is expressed in the kidney and parathyroid gland [11], and a deficiency of calcitriol decreases the expression of klotho [12]. Klotho-deficient mice have sarcopenia and decreased bone density. In a study of elderly humans, secreted klotho circulating in the blood was reported to affect lower limb function [13].

Based on the above, a decrease in calcitriol in the present study may have resulted in Type II muscle fiber atrophy, abnormalities in calcium metabolism, and decreased levels of anti-aging hormones, which may have had a significant impact on muscle weakness.

### 4.2. Clinical Implication and Future Research

The results of this study indicate that in the management of elderly patients with CKD, we need to fully consider the possibility of a low blood level of active vitamin D or calcitriol as a risk factor related to lower extremity muscle strength. In the future, it is necessary to verify whether vitamin D supplementation increases lower extremity muscle strength in pre-dialysis CKD patients.

### 4.3. Study Limitations

The present study has some limitations. The study population was relatively small, and consequently, we could not analyze sex- or age-related differences. Furthermore, we did not assess mechanisms, drugs, or drug reactions in the participants, nor did we evaluate individuals with comparable renal function using pair-matched analysis. We also did not evaluate surrogate parameters for sarcopenia, such as serum albumin or calf circumference, in relation to physical dysfunction, nor did we record the participants’ medications. There are several pharmaceuticals (e.g., corticosteroids, spironolactone, nifedipine, anticonvulsants, and rifampicin) that interact with vitamin D metabolism and can exacerbate vitamin D deficiency. These points will need to be evaluated in future trials.

## 5. Conclusions

In conclusion, these results suggest that a serum active vitamin D (calcitriol) level may be related to leg strength in older adults with pre-dialysis CKD. A low calcitriol concentration has been reported to induce Type II muscle fiber atrophy [4], and thus, as indicated in the present study, a low level of calcitriol may have affected lower extremity muscle strength.

An evaluation of muscle strength in elderly pre-dialysis CKD patients and encouraging them to exercise might be useful as minimum target goals for their rehabilitation.

## Figures and Tables

**Table 1:** Clinical characteristics of the patients.

Characteristic	Total
Number of patients	231
Age, years	76 (65−92)
Male, *n* (%)	159 (68.8)
Body mass index, kg/m^2^	24.1 ± 3.8
Calcitriol, pg/mL	32.5 ± 16.5
eGFR, mL/min/1.73 m^2^	21.5 (6.2−57.0)
Intact PTH, pg/mL	88 (6–607)
Phosphorus, mg/dL	3.7 ± 0.8
Calucium, mg/dL	9.1 (6.1−10.3)
Albumin, g/dL	3.9 ± 0.5
Knee extensor muscle strength, kgf/kg	0.44 ± 0.13

Abbreviations: eGFR, estimated glomerular filtration rate; PTH, parathyroid hormone.
